# Deciphering the immunogenic potential of wheat flour: a reference map of the salt-soluble proteome from the U.S. wheat Butte 86

**DOI:** 10.1186/s12953-020-00164-6

**Published:** 2020-08-01

**Authors:** Susan B. Altenbach, Han-Chang Chang, Annamaria Simon-Buss

**Affiliations:** 1 Western Regional Research Center, United States Department of Agriculture-Agricultural Research Service, Albany, CA USA; 2 Hamburg School of Food Science, Institute of Food Chemistry, University of Hamburg, Hamburg, Germany

**Keywords:** Albumins/globulins, Allergens, Celiac disease, Non-celiac wheat sensitivity, Wheat flour proteome, Mass spectrometry

## Abstract

**Background:**

Within the complex wheat flour proteome, the gluten proteins have attracted most of the attention because of their importance in determining the functional properties of wheat flour doughs and their roles in human health conditions such as celiac disease and food allergies. However, certain non-gluten proteins also trigger immunological responses but may be present in flour in low amounts or obscured by the more abundant gluten proteins in two-dimensional gels of total protein preparations.

**Methods:**

Non-gluten proteins were preferentially extracted from the flour with a dilute salt solution and separated by two-dimensional gel electrophoresis. Proteins in 173 gel spots were identified by tandem mass spectrometry after cleavage with trypsin or chymotrypsin. Transgenic wheat lines in which specific groups of gluten proteins were suppressed by RNA interference were used to estimate the amount of carry-over of gluten proteins in the salt-soluble protein fraction.

**Results:**

Fifty-seven different types of non-gluten proteins were identified, including 14 types that are known or suspected immunogenic proteins. The predominant proteins in 18 gel spots were gluten proteins. Some of these also contained non-gluten proteins. Analysis of the salt-soluble proteins from a transgenic line in which omega-1,2 gliadins were eliminated by RNA interference indicated that certain omega-1,2 gliadins were present in large amounts in the salt-soluble fraction and obscured relatively small amounts of beta-amylase and protein disulfide isomerase. In comparison, analysis of a transgenic line in which alpha gliadins were absent revealed that glyceraldehyde-3 phosphate dehydrogenase was a moderately abundant protein that co-migrated with several alpha gliadins.

**Conclusions:**

In this study, we constructed a proteomic map of the non-gluten protein fraction of wheat flour from the US wheat Butte 86 that complements a proteomic map of the total flour proteins developed previously for the same cultivar. Knowing the identities of low abundance proteins in the flour as well as proteins that are hidden by some of the major gluten proteins on two-dimensional gels is critical for studies aimed at assessing the immunogenic potential of wheat flour and determining which wheat proteins that should be targeted in future gene editing experiments to reduce the immunogenic potential of wheat flour.

## Background

Wheat flour proteins can be separated on the basis of solubility into the water/salt-insoluble gliadins and glutenins (gluten proteins) and the water/salt-soluble albumins and globulins (non-gluten proteins). The gluten proteins, comprising ~ 80% of the total protein, are a collection of ~ 60–80 different proteins that serve as the primary storage proteins of the grain while the non-gluten proteins are a diverse collection of different types of proteins that have metabolic, structural or defense functions. Much of the past research has focused on the gluten proteins because of their important role in determining the unique viscoelastic properties that give wheat flour its commercial value. These proteins are also of considerable interest because they are a major trigger of human health conditions, including celiac disease (CD), a chronic inflammatory disease of the small intestine that occurs in genetically susceptible individuals, and food allergies [1, 2]. The gliadins are primarily monomeric proteins that are divided into four groups termed alpha, gamma, omega and delta gliadins while the glutenins are polymeric proteins that consist of high molecular weight glutenin subunits (HMW-GS) and low molecular weight glutenin subunits (LMW-GS). Epitopes that stimulate T-cells in CD patients have been identified in proteins within most major classes of gluten proteins, although epitopes found in certain alpha gliadins and a subclass of omega gliadins, the omega-1,2 gliadins, have been found to be immunodominant [3, 4]. Gluten proteins in all major classes also react with IgE from patients with wheat allergy [5]. In particular, the omega-5 gliadins, another subclass of omega gliadins, have been found to trigger the serious food allergy wheat-dependent exercise-induced anaphylaxis that occurs in sensitized individuals when the ingestion of wheat is followed by physical exercise [6].

Non-gluten proteins also have been found to be immunogenic. In studies that examined cohorts of 28 and 60 patients with confirmed cases of wheat allergy, Battais et al. [5, 7] found that IgE from 67 and 72% of patients, respectively, reacted with proteins in the albumin/globulin fraction. Further studies using 1-D and 2-D immunoblots of albumin/globulin fractions demonstrated that a wide range of proteins often react with IgE from single patients and that the reactive proteins vary from individual to individual [8–11]. Non-gluten proteins likely to be allergenic are listed in the Allergome database (http://www.allergome.org/) and include beta amylase (Tri a 17), thioredoxin (Tri a 25), serpin (Tri a 33), triosephosphate isomerase (Tri a 31), peroxidase (Tri a peroxidase), chitinase (Tri a chitinase), peroxiredoxin (Tri a 32), glyceraldehyde 3-phosphate dehydrogenase (GAPDH) (Tri a 34), xylanase inhibitor (Tri a XI), dehydrin (Tri a 35), 9 kDa lipid transfer protein (Tri a 14) and a number of alpha amylase/trypsin inhibitors (AAI) (Tri a 28, Tri a 29, Tri a 30, Tri a 40). In addition, certain non-gluten proteins have been shown to be reactive with IgG and IgA antibodies from CD patients, suggesting a role for serpins, globulins, purinins, farinins and AAI in CD [12]. AAI also have been implicated in non-celiac wheat sensitivity (NCWS), a response to wheat ingestion that is distinct from CD and food allergies [13], while a globulin from wheat flour has been associated with the development of type 1 diabetes [14].

Because sensitivities to wheat are increasing, there is a need to identify clinically relevant proteins in wheat flour, associate specific flour proteins with particular types of allergies and sensitivities, and determine the frequencies of immunogenic responses to particular proteins. Two-dimensional immunoblot analysis is a powerful method for assessing the reactivity of patient sera to specific proteins in the flour [8, 10–12, 15–17]. The approach combines high resolution two-dimensional gel electrophoresis (2-DE), a robust, reproducible method for the both the separation and quantification of hundreds of proteins, with the identification of individual proteins by tandem mass spectrometry (MS/MS). However, 2-DE analyses of total protein preparations from wheat flour can be problematic because the more abundant gluten proteins overlap with low-abundance non-gluten proteins in some areas of the gels [18]. In a comprehensive analysis of total flour proteins, Dupont et al. [19] found MS/MS evidence for non-gluten proteins in 22% of 2-DE spots containing either LMW-GS or gliadins as the predominant proteins. In their study, LMW-GS overlapped with globulins, peroxidase, serpin and xylanase inhibitor; alpha gliadins overlapped with fructose bisphosphate aldolase, alcohol dehydrogenase and formate dehydrogenase; gamma gliadins overlapped with triticin and aspartic proteinase; and omega gliadins overlapped with beta-amylase. For this reason, salt-soluble protein fractions should also be considered for studies of immunogenic proteins. In this paper, we extracted proteins from flour from the US wheat Butte 86 with a dilute salt solution, separated the proteins by 2-DE, and identified proteins in 173 2-DE spots by MS/MS. The resulting proteomic map should make it possible to use 2-D immunoblots to rapidly identify and prioritize non-gluten proteins to be targeted in future gene editing experiments aimed at reducing the immunogenic potential of wheat flour.

## Methods

Proteins were extracted from wheat flour from the US spring wheat cultivar Butte 86 with a buffer containing 100 mM KCl as described in Hurkman and Tanaka [18], quantified by Lowry, and analyzed by 2-DE as described in detail in Dupont et al. [19]. In short, 50 mg flour was suspended in 200 μl of 50 mM Tris-HCl pH 7.8, 100 mM KCl, 5 mM EDTA and incubated for 5 min with intermittent vortex mixing. Samples were centrifuged at 4 °C at 14,500 x g for 15 min. The supernatant was collected and proteins were precipitated by the addition of 4 volumes of cold (− 20 °C) acetone. The pellet was rinsed with cold acetone, air-dried, and stored at -20 °C. After quantification, proteins were solubilized in urea buffer (9 M urea, 4% NP-40, 1% DTT, 2% ampholytes) for isoelectric focusing using capillary tube gels with a pI range of 3–10. NuPAGE 4–12% Bis-Tris protein gels (Life Technologies, Carlsbad, CA, USA) were used for the second dimension separation.

### LC-MS analysis

Protein spots were excised from replicate 2-D gels and placed in 96-well plates where they were reduced, alkylated and digested separately with trypsin (Sequencing Grade Modified Trypsin, Promega Corp., Madison, WI, USA) or chymotrypsin (Sequencing Grade Chymotrypsin, Promega Corp., Madison, WI, USA) using a DigestPro according to the directions of the manufacturer (Intavis, Koeln, Germany).

Plates containing peptides from digested gel spots were placed in the autosampler of a NanoLC 425 (Sciex, Framinham, MA, USA) that was interfaced by a nano-electrospray source to an Orbitrap Elite mass spectrometer (Thermo Scientific, San Jose, CA, USA). Ten μl fractions were loaded by the autosampler onto one of three PicoChip reverse phase columns (PicoChip with ReproSil-PUR C18, 10.5 cm, ID 75 μm, 3 μm, 120 Å, ReproSil-PUR-C18-AQ, New Objective Inc., Woburn, MA, USA). The positions of the columns were switched in front of the mass spectrometer with the PicoSlide system for Thermo LTQ/Orbi (New Objective Inc., Woburn, MA, USA). With this configuration, Column A is eluting, Column B is loading and Column C is washing, thereby saving time and reducing carryover between samples.

The loaded sample was washed with solvent A to remove salts and the column position was switched in front of the Orbitrap Elite mass spectrometer and eluted with a gradient of acetonitrile into the mass spectrometer. Solvent A was 5% in acetonitrile and Solvent B was 80% in acetonitrile, both solvents were 0.05% in formic acid. Gradient elution was at a flow rate of 250 nl per minute from 100% Solvent A to 35% Solvent B in 45 min. Peptides were detected in the Orbitrap set to scan a range from 200 to 2000 m/z at a resolution of 60,000. The 10 most intense peaks were subject to collision-induced dissociation (CID). The minimal signal threshold was set to 10,000. Dynamic exclusion with a repeat count of 2 was enabled for duration of 10 s. Normalized collision energy was set to 30%.

### Data analysis

The vendor specific .raw files were converted into .mgf files using MSConvert (ProteoWizard, Palo Alto, CA, USA). Spectral data was searched against a database containing all Triticeae sequences from NCBI downloaded on 02/07/2018, full-length Butte 86 sequences from Dupont et al. [19], full length Chinese Spring gluten protein sequences reported by Huo et al. [20, 21], and common MS contaminant sequences contained in the common Repository of Adventitious Proteins (cRAP) database (ftp://ftp.thegpm.org/fasta/cRAP/crap.fasta). Two search engines were used for analysis, Mascot (www.matrixscience.com) and XTandem! (https://www.thegpm.org/TANDEM/).

Results were loaded into Scaffold 4 (Scaffold_4.9.0, Proteome Software, Inc., Portland, OR, USA). The mass accuracy was set to 30 ppm on precursor level and 0.6 Da on MS/MS level. Scaffold summarized the Mascot and X!Tandem peptide hit results from the two different enzyme digestions (trypsin, chymotrypsin) with the MudPit analysis and compress data summarizing option.

Twenty-one spots that contained evidence of gluten proteins in this analysis were excised from replicate gels, digested with thermolysin (Promega Corp., Madison, WI, USA), and analyzed by MS/MS as above. These included spots 26, 27, 45, 54, 55, 60, 61, 80, 82, 83, 84, 87, 91, 101, 102, 104, 105, 106, 189, 190, and 191. Data from all three enzyme digestions were then combined in Scaffold. The predominant protein in each spot was deemed to be the protein for which the greatest number of unique peptides were identified and is reported in Table 1. All proteins identified in each spot are reported in Additional file 1. MS data for predominant proteins and proteins that contained at least half the number of unique peptides found in the predominant proteins are shown in Additional file 2. The mass spectrometry data have been deposited to the ProteomeXchange Consortium (http://proteomecentral.proteomexchange.org) via the PRIDE partner repository [23] with the dataset identifier PXD017260 and 10.6019/PXD017260.

**Table 1:** Predominant proteins identified by MS/MS in 2-DE protein spots from the KCl-soluble fraction of Butte 86 flour. All proteins had identification scores greater than 99% probability with at least 4 peptides as determined by Scaffold. Positions of spots in 2-DE are shown in Fig. 2. Identities of all proteins found in each spot are reported in Additional file 1

Spot Number	Predominant Protein	Accession #^a^	Source	# Unique peptides	# Spectra	% Coverage
1	pyruvate phosphate dikinase	XP_0201883631	*Ae. tauschii*	19	50	24
2	pyruvate phosphate dikinase	XP_0201883631	*Ae. tauschii*	19	83	21
3	pyruvate phosphate dikinase	XP_0201883631	*Ae. tauschii*	20	101	26
4	aconitate hydratase	XP_020176302	*Ae. tauschii*	13	31	15
5	no ID					
6	heat shock protein 101	AAD22629	*T.aestivum*	26	67	31
7	elongation factor 2	EMS59408	*T. urartu*	20	52	25
8	elongation factor 2	EMS59408	*T. urartu*	11	25	13
9	embryonic protein DC-8-like	XP_020196294	*Ae. tauschii*	14	50	24
10	embryonic protein DC-8-like	XP_020196294	*Ae. tauschii*	20	59	29
11	embryonic protein DC-8-like	XP_020196294	*Ae. tauschii*	17	65	31
12^b^	embryonic protein DC-8-like	XP_020196294	*Ae. tauschii*	30	85	38
13	embryonic protein DC-8-like	XP_020196294	*Ae. tauschii*	42	131	50
14	heat shock protein 90	ABG57075	*T.aestivum*	19	64	26
15	heat shock protein 70	XP_020151532	*Ae. tauschii*	43	209	48
16	heat shock protein 70	EMS51416	*T. urartu*	26	85	35
17	no ID					
18	beta-D-glucan exohydrolase	AAM13694	*T.aestivum*	25	84	34
19	beta-D-glucan exohydrolase	AAM13694	*T.aestivum*	24	60	33
20	beta-D-xylosidase	XP_020155854	*Ae. tauschii*	17	44	23
21^c^	glucose and ribitol dehydrogenase	pir T06212	*Hordeum vulgare*	7	19	28
22	no ID					
23	glucose-6-P isomerase	ABE41790	*T.aestivum*	22	67	37
24	glucose-6-P isomerase	ABE41790	*T.aestivum*	21	59	38
25	globulin 3A	AFM30909	*T.aestivum*	12	38	24
26^b,c^	beta-amylase	XP_020197275	*Ae. tauschii*	64	397	70
27^c,d^	beta-amylase	XP_020197275	*Ae. tauschii*	46	287	58
28	beta-amylase	EMS68884	*T. urartu*	21	83	37
29	beta-amylase	EMS68884	*T. urartu*	22	88	40
30	ketol-acid reductoisomerase	XP_020174215	*Ae. tauschii*	38	111	47
31	leucine aminopeptidase 2	EMS53149	*T. urartu*	26	61	47
32	atp1	ACA62607	*T.aestivum*	20	67	38
33	aldehyde dehydrogenase	AKE36953	*T.aestivum*	6	17	16
34	omega gliadin	AKB95614	*T. urartu*	18	34	51
35	enolase	XP_020163593	*Ae. tauschii*	33	135	78
36	enolase	XP_020163593	*Ae. tauschii*	55	164	89
37	enolase	BAJ85279	*Hordeum vulgare*	45	167	83
38^c^	enolase	BAJ85279	*Hordeum vulgare*	19	62	63
39	alanine aminotransferase	XP_020147857	*Ae. tauschii*	38	104	66
40	leghemoglobin reductase	XP_020185250	*Ae. tauschii*	35	139	69
41	cupincin	XP_020147625	*Ae. tauschii*	16	42	33
42	cupincin	XP_020147625	*Ae. tauschii*	30	90	47
43	cupincin	XP_020147625	*Ae. tauschii*	35	105	51
44	cupincin	XP_020147625	*Ae. tauschii*	33	151	47
45^d^	cupincin	XP_020147625	*Ae. tauschii*	35	192	53
46	cupincin	XP_020147625	*Ae. tauschii*	38	112	53
47	globulin-3A	AFM30909	*T.aestivum*	34	136	45
48	globulin-3A	AFM30909	*T.aestivum*	35	107	38
49	globulin-3A	AFM30909	*T.aestivum*	43	147	46
50	globulin-3A	AFM30909	*T.aestivum*	25	77	28
51	translation elongation factor 1 alpha	AAA34306	*T.aestivum*	25	109	44
52	globulin 3	ACJ65514	*T.aestivum*	5	13	8
53	globulin-3A	AFM30909	*T.aestivum*	25	83	28
54^d,e^	globulin-3A	AFM30909	*T.aestivum*	23	107	35
55^d,f^	LMW-GS	ACA63869	*T.aestivum*	27	84	53
56	globulin-3A	AFM30909	*T.aestivum*	7	23	14
57	LMW-GS	ABC84366	*T.aestivum*	28	54	60
58	LMW-GS	ABC84366	*T.aestivum*	9	14	29
59	ERBB-3 binding protein	XP_020191236	*Ae. tauschii*	11	53	29
60^d^	gamma gliadin	BU-gamma-5	*T.aestivum*	15	38	41
61^d^	gamma gliadin	BU-gamma5	*T.aestivum*	15	52	41
62	triticin	ACB41345	*T.aestivum*	15	49	26
63	isocitrate dehydrogenase	AMP82030	*T.aestivum*	11	41	27
64	serpin	EMS54555	*T. uratu*	34	102	68
65	serpin	CAB52709	*T.aestivum*	51	213	78
66	serpin	ACN59485	*T.aestivum*	35	109	66
67	serpin	ACN59485	*T.aestivum*	90	358	85
68	serpin	ACN59485	*T.aestivum*	55	162	78
69	serpin	ACN59484	*T.aestivum*	16	50	40
70	serpin	ACN59484	*T.aestivum*	25	75	48
71	serpin	AFC89429	*T.aestivum*	30	67	63
72	serpin	AFC89429	*T.aestivum*	87	268	86
73	serpin	AFC89429	*T.aestivum*	65	207	82
74	serpin	AFC89429	*T.aestivum*	11	36	32
75	glycosyltransferase 75	ADK56176	*T.aestivum*	7	18	22
76	serpin	CAA90071	*T.aestivum*	31	123	61
77	serpin	CAA90071	*T.aestivum*	31	61	61
78	serpin	EMS46390	*T. uratu*	39	115	67
79	alpha gliadin	BAM08450	*T.aestivum*	10	23	34
80^b,d,f^	alpha gliadin	CAY54134	*T.aestivum*	25	104	45
81^b^	ER molecular chaperone	AGN94840	*T.aestivum*	30	149	34
82^d,f^	gamma gliadin	BU-gamma-1	*T.aestivum*	29	164	50
83^d,f,g^	alpha gliadin	BU-alpha-23	*T.aestivum*	38	107	63
84^d^	LMW-GS	ABC84361	*T.aestivum*	17	48	33
85	no ID					
86^f^	LMW-GS	ACJ76984	*T. dicoccoides*	7	25	16
87^d,f^	gamma gliadin	AEA30015	*T.aestivum*	24	78	53
88	alpha gliadin	AAA96522	*T.aestivum*	11	24	42
89	peroxidase	XP_020147267	*Ae. tauschii*	38	173	53
90	peroxidase	AAM88383	*T.aestivum*	47	125	57
91^b,d,e^	peroxidase	AAM88383	*T.aestivum*	62	197	60
92	aldose 1 epimerase	XP_020180146	*Ae. tauschii*	9	24	22
93	globulin 3A	AFM30909	*T.aestivum*	19	57	25
94	63 kd globulin	XP-020163670	*Ae. tauschii*	24	88	27
95	farinin	XP_020147251 (BU-farinin-1)	*Ae. tauschii*	9	49	31
96	63 kd globulin	XP-020163670	*Ae. tauschii*	24	106	28
97	aspartic proteinase	XP_020157026	*Ae. tauschii*	9	27	19
98^g^	aspartic proteinase	XP_020157026	*Ae. tauschii*	5	13	13
99	cupincin	XP_020147625	*Ae. tauschii*	8	31	18
100	peroxidase	AAM88383	*T.aestivum*	13	36	34
101^b,d,h^	glyceraldehyde 3-P dehydrogenase	ALE18234	*T.aestivum*	49	164	65
102^b,d^	glyceraldehyde 3-P dehydrogenase	ANW11922	*T.aestivum*	79	230	92
103^b,d^	glyceraldehyde 3-P dehydrogenase	ALE18234	*T.aestivum*	37	85	68
104^b,d^	fructose bisphosphate aldolase	CDM85265	*T.aestivum*	60	197	73
105^b,d^	glyceraldehyde 3-P dehydrogenase	ABQ81648	*T.aestivum*	56	208	73
106^b,d,f^	alpha gliadin	CS-alpha-A1	*T.aestivum*	70	215	77
107	malate dehydrogenase	XP_020196362	*Ae. tauschii*	6	17	17
108	guanine nucleotide binding protein	CDM83867	*T.aestivum*	11	23	40
109	malate dehydrogenase	XP_020157252	*Ae. tauschii*	44	149	73
110	malate dehydrogenase	AAT64932	*T.aestivum*	21	53	54
111	desiccation-related protein	XP_020169835	*Ae. tauschii*	16	37	35
112	desiccation-related protein	XP_020169835	*Ae. tauschii*	11	34	31
113	lactoylglutathione lyase	XP_020185375	*Ae. tauschii*	13	38	41
114	lactoylglutathione lyase	XP_020185375	*Ae. tauschii*	51	149	79
115	lactoylglutathione lyase	XP_020185375	*Ae. tauschii*	16	54	44
116	lactoylglutathione lyase	EMS56223	*T. uratu*	7	19	21
117	glucose and ribitol dehydrogenase	XP_020188730	*Ae. tauschii*	16	41	46
118	glucose and ribitol dehydrogenase	XP_020188730	*Ae. tauschii*	28	107	61
119	glucose and ribitol dehydrogenase	XP_020188730	*Ae. tauschii*	31	127	67
120	glucose and ribitol dehydrogenase	XP_020188730	*Ae. tauschii*	29	101	65
121	proteosome subunit alpha type 1	BAJ85935	*Hordeum vulgare*	10	28	39
122	glucose and ribitol dehydrogenase	BAJ85825	*Hordeum vulgare*	15	64	39
123	dehydrin	AOM63239	*T.aestivum*	12	29	61
124	purinin	ADA62372 (BU-purinin-3)	*T.aestivum*	14	105	40
125	19 kd globulin	XP_020162707	*Ae. tauschii*	12	65	61
126	oil body associated protein	XP_020196637	*Ae. tauschii*	24	74	81
127	19 kd globulin	XP_020162707	*Ae. tauschii*	9	62	50
128	xylanase inhibitor	AGN71004	*T.aestivum*	34	127	64
129	globulin-1	ABG68034	*T.aestivum*	12	55	46
130	chitinase	XP_020181917	*Ae. tauschii*	19	108	41
131	globulin	AAR95703	*T. turgidum*	11	65	50
132	globulin	AAR95703	*T. turgidum*	10	72	50
133	tritin	BAA02948	*T.aestivum*	53	316	75
134	farinin	XP_020147251 (BU-farinin-1)	*Ae. tauschii*	7	35	21
135	secretory protein	XP_020161391	*Ae. tauschii*	9	23	38
136	secretory protein	XP_020161391	*Ae. tauschii*	5	14	29
137	cyclophilin A	AAK49426	*T.aestivum*	14	84	57
138	chitinase	XP_020181917	*Ae. tauschii*	15	28	32
139	chitinase	AAX83263	*T.aestivum*	20	73	48
140	AAI	prf 1207200A (WASI BU-1)	*T.aestivum*	39	234	82
141	AAI	prf 1207200A (WASI BU-1)	*T.aestivum*	14	72	51
142	AAI	prf 1207200A (WASI BU-1)	*T.aestivum*	31	112	71
143	peroxiredoxin	ACE82290	*T.aestivum*	21	91	67
144	1-cys peroxiredoxin	AAQ74769	*T.aestivum*	21	73	65
145	purinin	EMS62569 (BU-purinin-1)	*T. urartu*	22	98	68
146	purinin	EMS62569 (BU-purinin-1)	*T. urartu*	9	37	52
147	triosephosphate isomerase	CDM81250	*T.aestivum*	31	102	70
148	oil body associated protein	XP_020147075	*Ae. tauschii*	30	103	84
149	late embryogenesis abundant protein	XP_020193122	*Ae. tauschii*	10	22	55
150	late embryogenesis abundant protein	XP_020193122	*Ae. tauschii*	14	39	62
151	late embryogenesis abundant protein	BAK04960	*Hordeum vulgare*	6	16	28
152	glutathionine-S-transferase	XP_020196644	*Ae. tauschii*	15	57	49
153	glutathionine-S-transferase	XP_020196644	*Ae. tauschii*	51	214	60
154	glutathionine-S-transferase	XP_020196644	*Ae. tauschii*	21	65	53
155	lysosomal thiol reductase	XP_020155412	*Ae. tauschii*	15	73	52
156	nucleoside diphosphate kinase	EMS63724	*T. urartu*	5	16	41
157	AAI	AAR10959 (WTAI-CM3 BU-1)	*T.aestivum*	37	388	74
158	no ID					
159	AAI	CAI84642 (WDAI BU-1)	*T.aestivum*	8	24	66
160	no ID					
161	no ID					
162	no ID					
163	ubiquitin	AAP50253	*T.aestivum*	10	56	83
164	AAI	AAV39514 (WDAI BU-1)	*T.aestivum*	26	244	100
165	AAI	AAV39517 (WDAI BU-1)	*T.aestivum*	22	101	88
166	AAI	sp P16851 (WTAI-CM2 BU-1)	*T.aestivum*	24	107	74
167	superoxide dismutase	AFF27607	*T.aestivum*	18	61	66
168	globulin-1	EMS62417	*T. urartu*	12	53	15
169	AAI	ABO45967 (WMAI BU-1)	*T.aestivum*	25	135	99
170	AAI	ABI54484 (WDAI BU-3)	*T. dicoccoides*	14	58	96
171	AAI	ABO45967 (WMAI BU-1)	*T.aestivum*	19	116	98
172	AAI	AAV91972 (WDAI BU-4)	*T.aestivum*	37	198	100
173	AAI	ACM41418 (WTAI-CM16 BU-1)	*T. macha*	34	228	80
174	AAI	CAA42453 (WTAI-CM-17 BU-1)	*T.aestivum*	25	133	77
175	AAI	CAA42453 (WTAI-CM-17 BU-1)	*T.aestivum*	13	59	50
176	AAI	ABO45967 (WMAI BU-1)	*T.aestivum*	4	17	36
177	AAI	sp P16851 (WTAI-CM2 BU-1)	*T.aestivum*	7	25	44
178	homocysteine methyltransferase	EMS51950	*T. urartu*	20	76	27
189^d,f^	alpha gliadin	BU-alpha-3	*T.aestivum*	15	44	35
190^d,e^	alpha gliadin	BAM08458	*T.aestivum*	23	73	50
191^b,d^	alpha gliadin	BU-alpha-4	*T.aestivum*	19	53	46

^a^ Accession numbers beginning with BU are from Butte 86 and CS are from Chinese Spring. All other accession numbers are from NCBI. The Butte 86 accession is shown in parentheses in cases where a sequence from NCBI is identical to a sequence from Butte 86. Gliadin, purinin and farinin sequences from Butte 86 are from [19]. AAI sequences from Butte 86 are from [22]

^b^ a gluten protein with less than half the number of unique peptides found in the predominant protein was also identified in spot

^c^ also contains an omega gliadin

^d^ replicate sample was digested with thermolysin and analyzed by MS/MS

^e^ also contains a LMW-GS

^f^ also contains a non-gluten protein

^g^ also contains a gamma gliadin

^h^ also contains an alpha gliadin

Flour from transgenic lines missing specific gluten proteins was used to further evaluate spots containing both gluten proteins and non-gluten proteins. Transgenic lines were produced previously using RNA interference. Transgenic line SA-30-118a-5 is missing all omega-1,2 gliadins [16] while transgenic line SA-35a-124j is missing all alpha gliadins [17].

## Results

**Fig. 1:**
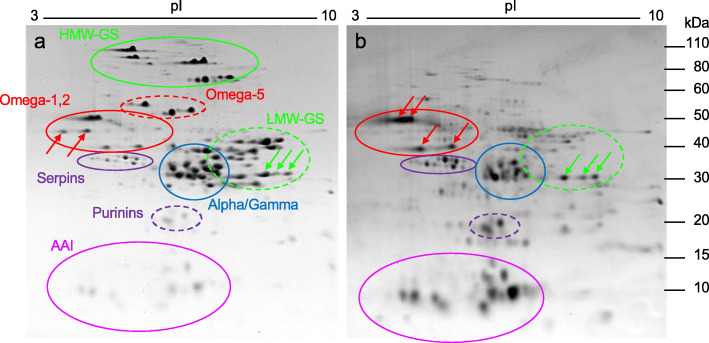
2-DE separation of total SDS-extracted proteins (**a**) and KCl-soluble proteins (**b**) from Butte 86 wheat flour. The positions of major protein groups based on the MS identifications of proteins in Dupont et al. [19] are indicated in red (omega-1,2 gliadins), dashed red (omega-5 gliadins), blue (alpha and gamma gliadins), green (HMW-GS), dashed green (LMW-GS), purple (serpins), dashed purple (purinins), or magenta (AAI) ovals. Red arrows in panel a point to omega-1,2 gliadins that are found in the total protein fraction but not in the KCl-soluble fraction while red arrows in panel b point to proteins likely to be omega-1,2 gliadins that partition into the KCl-soluble fraction. Green arrows point to spots in which the predominant proteins were identified as LMW-GS in the total protein fraction, but peroxidases in the KCl-soluble fraction. The position of size markers in kDa are shown on the right

KCl-soluble proteins from Butte 86 flour were separated by 2-DE and the pattern was compared to that of a total SDS protein extract (Fig. 1). The most abundant proteins in the total protein extract, representing 125 of 233 2-DE spots identified previously by MS/MS [19], were the gluten proteins; the HMW-GS and LMW-GS (shown in green), the alpha and gamma gliadins (shown in blue) and the omega gliadins (shown in red) (Fig. 1a). Several groups of non-gluten proteins, including serpins, purinins and alpha-amylase/trypsin inhibitors (AAI), were well-separated from the major gluten proteins but of considerably lower abundance. In comparison, the most abundant proteins in the KCl-soluble fraction (Fig. 1b) were located in the AAI, omega-1,2 gliadin and alpha and gamma gliadin regions of the gel. Additionally, proteins in the serpin and purinin regions were notably more abundant in the KCl-soluble fraction than in the total protein fraction.

**Fig. 2:**
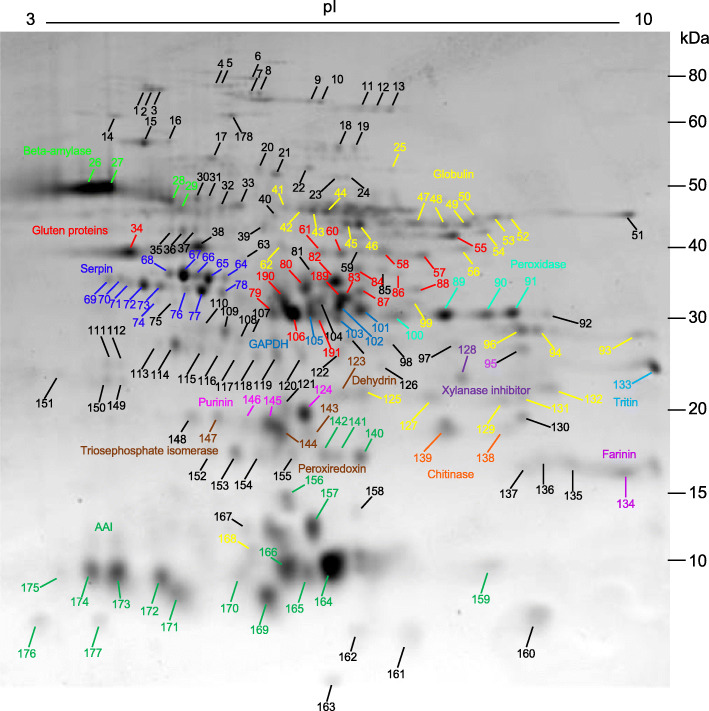
Identification of proteins in a KCl-soluble fraction of wheat flour. Spots in which the predominant proteins were gluten proteins are labeled in red. Spots in which the predominant proteins were non-gluten proteins that are either known allergens or likely to be immunogenic are labeled in bright green (beta-amylase), dark blue (serpins), medium blue (glyceraldehyde-3-phosphate dehydrogenase (GAPDH), yellow (globulins), teal (peroxidase), magenta (purinins), orange (chitinase), purple (xylanase inhibitor), light blue (tritin), light purple (farinin), brown (dehydrin, peroxiredoxin, triosephosphate isomerase), or green (AAI). Protein identifications can be found in Table 1 and Additional file 1

One-hundred-eighty-one spots were excised from 2-D gels of the KCl-soluble proteins and analyzed by MS/MS (Fig. 2). One hundred seventy-three of the spots yielded valid identifications. MS/MS coverage of the proteins ranged from 8 to 100% with an average of 49%. Sixty-one different protein types and 111 sequences were identified as the predominant proteins in these spots (Table 1). The predominant proteins in 26 spots were globulins (Fig. 2). Nine different globulin sequences were identified. Seventeen spots were identified as AAI and 11 different sequences were found. Fourteen spots were serpins and seven different sequences were identified. In addition, the predominant proteins in multiple spots were identified as glucose and ribitol dehydrogenase (6), embryonic protein DC-8 (5), beta-amylase (4), enolase (4), GAPDH (4), lactoylglutathione lyase (4), peroxidase (4), chitinase (3), glutathione-S-transferase (3), late embryogenesis abundant protein (3), malate dehydrogenase (3), pyruvate phosphate dikinase (3), and purinin (3). Forty other non-gluten protein types were identified as the predominant proteins in either one or two 2-DE spots (Table 1). Only eight of these were identified previously in the total protein fraction [19].

In some cases, gluten proteins were separated effectively from non-gluten proteins on the basis of solubility. For example, peroxidase was the predominant protein in three moderately abundant spots (89, 90, 91) in the LMW-GS region of the gel from the KCl-soluble fraction (green arrows in Figs. 1b and 2). In the total protein fraction of Dupont et al. [19], the predominant proteins in these three spots were LMW-GS (indicated with green arrows in Fig. 1a). The absence of most LMW-GS in the KCl-soluble fraction also made it easier to visualize many of the minor globulins that are found in this region of the gel (labeled in yellow in Fig. 2).

Despite the fact that most gluten proteins are not readily soluble in salt solutions, gluten proteins were the predominant proteins identified in 18 spots in the KCl fraction (Fig. 2). Eight spots were alpha gliadins, four were gamma gliadins, one was an omega gliadin and five were LMW-GS (Table 1). Fourteen of these spots also contained non-gluten proteins (34, 55, 60, 61, 80, 82, 83, 84, 86, 87, 106, 189, 190, 191). Gluten proteins also were detected as minor components of 14 spots (Table 1, Additional file 1).

Four major spots (26, 27, 34, 38) in the omega-1,2 gliadin region of the gel were confounding (indicated with red arrows in Fig. 1b, Fig. 2). The predominant proteins identified in spots 26 and 27 were beta-amylase (Table 1). However, protein disulfide isomerase and omega-1,2 gliadin BAN29067 also were identified in these spots (Additional file 1). Spot 34 was identified as omega-1,2 gliadin AKB95614 whereas spot 38 contained both enolase and omega-1,2 gliadin AKB95614. Interestingly, two other spots previously identified as omega-1,2 gliadins (shown with red arrows in Fig. 1a) did not appear in the KCl-soluble fraction even though their sequences were very similar to the omega-1,2 gliadins found in spots 26 and 27 [16].

A number of spots in the region of the gel containing alpha and gamma gliadins also were problematic. Three minor spots in this region contained only alpha gliadins (79, 88, 191) while two minor spots contained only gamma gliadins (60, 61). However, five moderately abundant spots contained both an alpha gliadin as the predominant protein and GAPDH (83, 106, 189, 190, 191). Additionally, the predominant proteins in four other spots were GADPH, but MS/MS evidence also pointed to the presence of alpha gliadins in these spots (101, 102, 103, 105) (Fig. 2, Additional file 1).

**Fig. 3:**
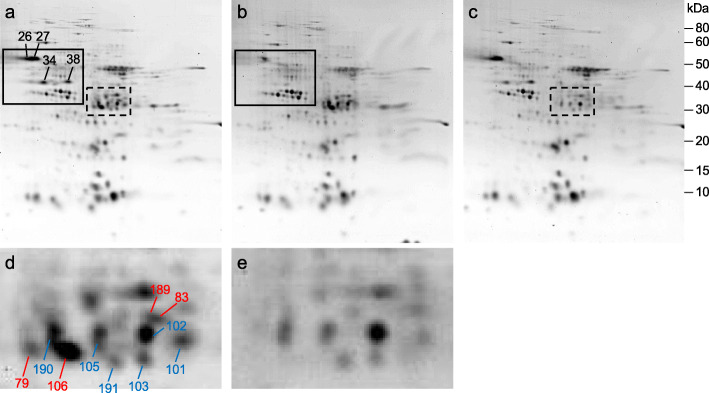
KCl-soluble flour proteins in Butte 86 (**a**) and transgenic lines SA-30-118a-5 (**b**) and SA-35a-124j (**c**). The solid box highlights regions of the gels containing the omega-1,2 gliadins while the dashed box highlights regions of gels containing the alpha gliadins. The alpha gliadin regions from Butte 86 and SA-35a-124j were enlarged in panels (**d**) and (**e**), respectively. Spots labeled in red in panel d were significantly reduced or absent in SA-35a-124j and are predominantly alpha gliadins that partition into the KCl fraction. Spots labeled in blue in panel (**d**) were also found in the KCl fraction of SA-35a-124j and contain mostly GAPDH

To estimate the amounts of different gluten proteins that were carried over into the KCl-soluble protein fraction, we examined the profiles of flour proteins from transgenic lines in which the expression of either the omega-1,2 gliadins or the alpha gliadins was suppressed by RNA interference [16, 17]. Detailed proteomic analyses of total protein fractions from flour of the transgenic lines revealed that all omega-1,2 gliadins were absent in line SA-30-118a-5 [16] while all alpha gliadins were absent in line SA-35a-124j [17]. In 2-D gels of the KCl-soluble protein fraction, spots 26, 27, 34, 38 containing omega-1,2 gliadins were major spots in the non-transgenic flour (Fig. 3a). Spot 26 was significantly reduced while other spots were not apparent in transgenic line SA-30-118a-5 (Fig. 3b), suggesting that significant amounts of certain omega-1,2 gliadins partition into the KCl-soluble fractions and that beta-amylase and protein disulfide isomerase are low abundance proteins. Within the alpha gliadin region of the gel (Fig. 3a, c), several spots in which either alpha gliadins (79) or alpha gliadins and GAPDH (83, 106, 189) were identified in the KCl fraction from Butte 86 were not apparent in transgenic line SA-35a-124j, suggesting that alpha gliadins comprise the bulk of the protein in these spots. Several other spots containing both alpha gliadin and GAPDH were present in similar amounts in Butte 86 and the transgenic line (101, 102, 103, 105, 190, 191), suggesting that most of the protein in these spots is GAPDH and that this protein is fairly abundant in the KCl fraction. Interestingly, GADPH was not identified in the total protein fraction in the Dupont et al. (2011) study [19].

## Discussion

A number of non-gluten proteins that are abundant in the KCl-soluble protein fraction are well-separated from the major gluten proteins and can be identified unambiguously by MS/MS in a total SDS protein extract of Butte 86 flour [19]. These include the serpins, purinins and AAI. However, other non-gluten proteins are overshadowed by the more abundant gluten proteins. In 2-DE gels of total protein extracts, beta-amylases are hidden by omega-1,2 gliadins, GAPDHs are masked by alpha gliadins, and peroxidases and some globulins are obscured by LMW-GS. In this study, non-gluten proteins were preferentially extracted from wheat flour using KCl and 57 different types of non-gluten proteins were identified, including 14 types that are known or suspected immunogenic proteins. Although most gluten proteins are insoluble in KCl, we observed some carry-over of the abundant gluten proteins in the KCl fraction. This was not unexpected and, in contrast to other studies [8–11], was likely noted because protein spots were digested separately with trypsin and chymotrypsin before analysis by MS/MS, thereby facilitating the identification of both non-gluten proteins that are readily digested with trypsin and gluten proteins that have few tryptic peptide cleavage sites. For example, 276 tryptic peptides were identified for beta-amylase in spot 27 and 62 tryptic peptides were identified for enolase in spot 38. The omega-1,2 gliadins in these two spots did not yield any tryptic peptides, but were identified on the basis of 81 and 41 chymotryptic peptides, respectively. Likewise, 72 tryptic peptides were identified for GAPDH in spot 101. Although the alpha gliadin in the same spot did not yield any tryptic peptides, it was identified by 24 chymotryptic peptides (Additional file 2). In general, protein spots that contained either gamma gliadins or LMW-GS were relatively minor while spots containing certain omega-1,2 gliadins or alpha gliadins were quite prominent in the KCl-soluble fraction. Interestingly, two spots found in the omega-1,2 gliadin region of the total protein fraction but not in the KCl-soluble fraction were identified previously as omega-1,2 gliadins that contain single cysteine residues [16]. These cysteine residues likely enable the proteins to be incorporated into glutenin polymers, rendering them insoluble in aqueous solutions even though their sequences are otherwise quite similar to omega-1,2 gliadins that partition into the KCl fraction. Indeed, in past experiments, these omega-1,2 gliadins were accumulated preferentially in the polymer fractions of Butte 86 wheat flour [24].

The availability of transgenic lines missing either the omega-1,2 gliadins or the alpha gliadins made it possible to estimate the abundance of specific non-gluten proteins that overlap with gluten proteins in the KCl-soluble fraction. These analyses demonstrated that beta-amylase and protein disulfide isomerase are low abundance proteins while GAPDH is a protein of moderate abundance.

Di Francesco et al. [25] recently used a shotgun approach to characterize the KCl-soluble proteins from old and modern durum wheats. In their study, proteins were prepared from mature kernels rather than milled flour and the protein extracts were digested only with trypsin. Analysis of the resulting spectral data used a database of protein sequences from *Triticum, Oryza, Hordeum, Avena, Secale, Maize* and *Brachypodium* downloaded from the UniProt database. Interestingly, only 21% of identified proteins were from *Triticum*, 10.8% from *Hordeum* and 1% from *Secale*. In the current study, proteins were digested individually with either trypsin or chymotrypsin and spectra were searched against a database containing all *Triticeae* sequences from NCBI along with a selection of sequences from Chinese Spring and Butte 86. Not surprisingly, many of the same types of proteins, including likely allergens, were identified in both studies. However, it is curious that Di Francesco et al. identified only one globulin in their study while nine different globulin sequences were identified in 26 2-DE spots in the current study. Likewise, Di Francesco et al. identified only three different serpins, while seven different sequences in 14 spots were found in the current study. They did, however, report a number of alpha and gamma gliadins as well as HMW-GS and LMW-GS in the KCl fraction, confirming the carryover of gluten proteins into this fraction that was also observed in our analysis. It is likely that they would have identified more gluten proteins in this fraction if proteins had been digested with both trypsin and chymotrypsin prior to MS analysis.

## Conclusions

The proteomic map of KCl-soluble flour proteins generated in this study complements the map of total flour proteins from Butte 86 described by Dupont et al. [19] and serves as a reference map that, combined with 2-D immunoblots using sera from well-characterized patients, should provide new insight into non-gluten proteins that are responsible for immunogenic responses to wheat flour and lead to a better understanding of the complement of proteins associated with different types of allergies. Transgenic lines missing specific groups of gluten proteins should be of value in confirming whether reactivity with patient antibodies is due to non-gluten proteins or to gluten proteins that are carried over into the KCl-soluble fraction. This information is critical for future efforts to develop wheat with reduced immunogenic potential.

## Supplementary information

**Additional file 1.** Proteins identified by MS/MS in 2-DE protein spots from the KCl-soluble fraction of Butte 86 flour. The positions of the spots are shown in Figure 2. For each spot, the predominant protein and all proteins that contained at least half the number of unique peptides as the predominant protein are highlighted in yellow. Proteins that contained less than half the number of unique peptides found in the predominant protein are highlighted in blue. Gluten proteins that contained less than half the number of unique peptides found in the predominant protein are highlighted in green.

**Additional file 2.** MS/MS data for individual protein spots shown in Figure 2. Scaffold data for all proteins for which the number of unique peptides was equal to or greater than half the number assigned to the predominant protein are shown.

## Data Availability

The dataset generated in this study has been deposited in the ProteomeXchange Consortium (http://proteomecentral.proteomexchange.org) via the PRIDE partner repository.

## Abbreviations

2-DE: Two-dimensional gel electrophoresis
AAI: Alpha amylase/trypsin inhibitors
CD: Celiac disease
GAPDH: Glyceraldehyde 3-phosphate dehydrogenase
HMW-GS: High molecular weight glutenin subunit
LMW-GS: Low molecular glutenin subunit
MS/MS: Tandem mass spectrometry
NCWS: Non-celiac wheat sensitivity
